# Udder Morphometry and Its Relationship with Intramammary Infections and Somatic Cell Count in Serrana Goats

**DOI:** 10.3390/ani10091534

**Published:** 2020-08-31

**Authors:** Gisele Margatho, Hélder Quintas, Vicente Rodríguez-Estévez, João Simões

**Affiliations:** 1 Animal and Veterinary Research Centre (CECAV), University of Trás-os-Montes e Alto Douro, Quinta de Prados, 5370-801 Vila Real, Portugal; 2 Vasco da Gama Research Group (CIVG), Vasco da Gama University School (EUVG), 3020-210 Coimbra, Portugal; 3 Mountain Research Centre (CIMO), School of Agriculture, Polytechnic Institute of Bragança (IPB), Campus de Santa Apolónia, 5300-253 Bragança, Portugal; helder5tas@ipb.pt; 4 Department of Animal Production, University of Córdoba, Campus de Rabanales, 14071 Córdoba, Spain; vrestevez@uco.es

**Keywords:** goats, mastitis, morphometry, udder

## Abstract

**Simple Summary:**

The present study aimed to characterize the external morphological traits of the mammary gland and their relationships with the presence of intramammary infection and the somatic cell count (SCC) of Serrana goats, Transmontano ecotype. Bifurcated pendular udders, with vertical loose teats and located close to each other, are more likely to have intramammary infection, and have the highest SCC. The udder shape, symmetry, degree of suspension and degree of separation parameters have shown to be significantly different depending on SCC.

**Abstract:**

The external morphological traits of the mammary gland, and their relationships with somatic cell count (SCC) and the presence of intramammary infection (IMI), were studied in 30 Serrana goats, Transmontano ecotype. Globular-shaped udders were the most predominant, with slightly separated and symmetrical halves, presenting some degree of suspension. Funnel-shaped teats were the most prevalent shape with an opening of 120° to 160° degrees. Significant differences were observed between healthy group and the coagulase negative staphylococci (CNS)-infected group for udder cleft, teat perimeter and distance between teats parameters; and between healthy group and CNS or *Staphylococcus aureus* groups for degree of separation, teat shape and udder shape (*p* < 0.05). The udder shape, symmetry, degree of suspension and degree of separation parameters showed to be different depending on SCC (*p* < 0.05). The udder perimeter and udder depth traits showed differences between the lowest and the middle SCC group. We concluded that bifurcated pendular udders, with vertical loose teats and located close to each other, are more likely to have IMI, and have the highest SCC. The inclusion in breeding programs of certain mammary conformation traits would not only help to improve milk production, but would also decrease the susceptibility to IMI of the herd.

## 1. Introduction

The Serrana goat breed, Transmontano ecotype, is usually raised in small flocks in the traditional extensive pastoralism system in the northeast region of Portugal. It has a great socio-economic impact, contributing to the settlement of populations in rural areas and being the main economic resource for many families. Serrana goats are the major Portuguese local breed. They are a medium-sized, dual-purpose (meat and milk) goat reared under a pastoralist system in small farms normally with an average of 80–100 goats. For a description of this breed and production system [1], see Sociedade Portuguesa de Ovinotecnia e Caprinotecnia [2].

A simple and inexpensive indicator of potential individual milk yield is the udder phenotypic characteristics, which have an adequate genetic and also environmental variation [3,4,5]. Udder traits also seem to be related to an individual’s resistance to intramammary infections (IMI) [6,7], which are the main cause of decreased milk quality and quantity, with consequent high economical losses in small ruminant farms [8]. Conformation traits are currently being analyzed and used as criteria for goat genealogical registration, based mainly on the aesthetic aspect, with few corroborating studies that relate these conformations to udder health [9,10]. It is known that certain mammary gland conformations have a greater propensity for post milking wounds, which serve as a gateway to pathogenic microorganisms and possible intramammary infection [11]. More knowledge about the relationship between udder conformation characteristics and mastitis can be utilized in the design of breeding programs to improve milk ability and udder health. It seems important, though, to establish a correlation between udder conformation and IMI, allowing for the selection of higher mastitis resistance in dairy goats [12,13].

The main aim of the present study was to evaluate the associations between the morphology of the external mammary gland and the presence of intramammary infections, by using independently the conventional bacteriology and somatic cell count (SCC) diagnostic methods.

## 2. Materials and Methods

The study was approved by the Ethics Committee of IPB, the Instituto Politécnico de Bragança (process number RI001-2019/02376).

### 2.1. Animal and Management Conditions

The experiment was conducted with 30 goats from the portuguese Serrana breed, Transmontano ecotype, from one experimental flock, of Instituto Politécnico de Bragança (IPB), and another from Escola Profissional de Agricultura e Desenvolvimento Rural de Carvalhais/Mirandela (EPA), both in the region of Trás-os-Montes in the northeast of Portugal. All goats were selected according to the posterior bacterial/fungal results. Only goats with halves infected by a single bacterial group, genus or species, as well yeasts (infected groups) or without infection (control group), were considered. The animals between their 1st and 8th lactation, weighing 31–52 kg, were fed with commercial balanced ration and water ad libitum. Of the animals, 63% were in the early stages of lactation (2nd month), and 36% were on the 6th month. The goats were milked one time per day using a mechanical milking machine, but some of them were suckling kids. The environmental variables, temperature and humidity were recorded at the measurement time, inside the barn, with the use of an Extech^®^ HT30 m (Nashua, NH, USA). The mean air temperature and air humidity was 14 °C and 68%, respectively.

### 2.2. Udder Conformation

**Figure 1:**
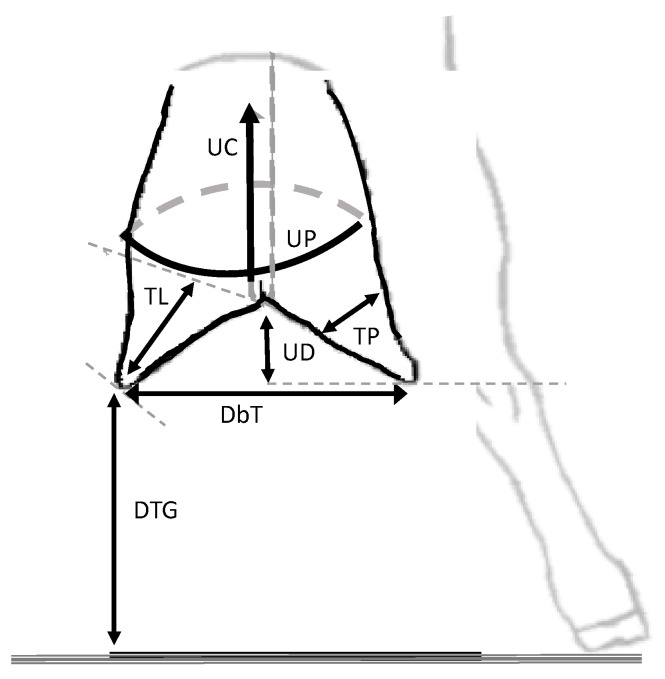
Udder physical conformation measurements. UP = Udder perimeter; TP = teat perimeter; DBT = distance between teats; TL = Teat length; UD = Udder depth; TDG = Teat distance to the ground; UC = Udder cleft.

In order to evaluate udder conformation, the animals were kept in a standing position on slope-free ground; measurements were taken following the parameters (Figure 1) described by [14], adapted by [11].

The physical measurements taken using a tape measure on the udder were udder perimeter (UP), as the horizontal measurement of the udder performed in the middle portion; the udder cleft (UC), as the vertical measurement of the udder from the medial cranial insertion to the medial caudal insertion, passing through the region of the medial suspensory ligament; and udder depth (UD), measured between the most ventral point of the udder floor to the ground, subtracting the measurement between the tibiotarsal joint and the ground. The measurements taken on the teats were the following: teat length (TL), the measurement from the base to the teat’s end; teat perimeter (TP), a horizontal measurement performed in the middle portion of the teat; distance between teats (DBT), as the distance between sphincters of the teats; and the teat distance to the ground (TDG), as the minimum measured distance between teat end and the ground.

**Figure 2:**
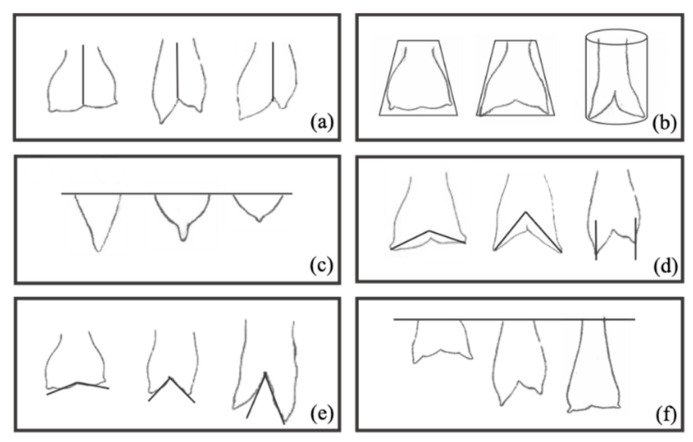
Morphological characterization of the sample (Serrana goats); Parameters: Symmetry (**a**), Udder shape (**b**), Teat Shape (**c**), Teat angle (**d**), Degree of separation (**e**), Degree of suspension (**f**).

A subjective evaluation of mammary morphology traits was preliminarily made based on visual appraisal, without resorting to measurements [14,15,16]. For the subjective morphological evaluation, six parameters of the mammary gland were established, and the main profiles were defined according to our sample. All measurements and subjective evaluations of the mammary gland were carried out by the same person. The udders were classified by Shape (Ushape) (Globular (1), Pear-shaped (2), Cylindrical pendulous (3)), Symmetry (Symm) (Symmetrical (0), moderate (1) or Asymmetric (2)), Degree separation (Dsep) (Slight (1), Moderate (2) or Severe (3)) and Degree of suspension (Dsusp) (Extremely snug (1), Intermediate (2), Pendulous (3) or Extremely loose (4)) [17,18]. Similarly, the teat shape (Tshape) was classified as Funnel (1), Bottle (2) or Balloon (3) [11], and teat angle (Tangle) (orientation) as 160°–180° (1), 120°–160° (2) or 90°–120° (3) degrees (Figure 2).

### 2.3. Milk Sampling, Somatic Cell Counts and Microbial Identification

Milk samples were obtained aseptically from each gland of each animal. Before sampling, the teat ends were cleaned with 70% alcohol, the first strips of milk were discarded and approximately 20 mL of milk from each gland of each animal was collected twice in sterile tubes for somatic cell count and bacteriological status. Samples were refrigerated in an ice box and transported immediately to the laboratory (ALIP—Associação Interprofissional do Leite e Lacticínios), then analyzed 24 h after collection. Somatic cell counts were determined using the Fossomatic^TM^ FC equipment (Foss, HillerØd, Denmark) [19], calibrated with bovine milk somatic cell standard. Specific pathogen identification from both mammary glands was performed using the automatized VITEK^®^2 Compact (Biomérieux) commercial method [20].

### 2.4. Statistical Analysis

Variables normality was previously analyzed using the Shapiro–Wilk test statistic. All the values, including SCC after log10-transformation, did not present a normal distribution, and non-parametric tests were used. The halves were considered the unit level. The data of the measured parameters were tested for correlation significance with the Spearman correlation coefficient, in SPSS^©^ for Mac (version 26.0.0.0, SPSS Inc., Chicago, IL, USA). A correlation was considered strong if 0.700 < |*r*| < 1.000, and weak if 0.0 < |*r*| < 0.400. The effect of bacteriological status and the effect of SCC on udder morphological traits have been studied using the Kruskal–Wallis test for independent samples, with subsequent Kruskal–Wallis 1-way ANOVA pair-wise comparisons (K samples), in SPSS^©^ for Mac (version 26.0.0.0, SPSS Inc., Chicago, IL, USA). Differences were considered statistically significant at *p* ≤ 0.05.

## 3. Results

### 3.1. Udder Conformation

The measurable udder characteristics were reported in Table 1 and shown to be closely related to each other (Table 2). Udder parameters are moderately correlated with teat parameters. The UP presents a moderate positive correlation with teat parameters, such as TP, TL and DBT, a high positive correlation with UC and a negative correlation with UD and TDG; i.e., as the udder gets wider the teats also increase in size, and the distance between them increases, consequently increasing the length and cleft value and decreasing the distance to the ground.

The teat parameters (TP, TL, DBT) seem to increase together with the increase in the size (perimeter and length) of the udder. Its increase represents a closer proximity to the ground. UD values represent the measurement between the most ventral point of the udder floor to the ground, subtracting the measurement between the tibiotarsal joint and the ground, meaning that the bigger this positive difference is, the more attached the udder is. Therefore, when the UP, UC, TP, DBT and TL increase, the udder is going to be deeper/longer. UC is the vertical measurement of the udder from the cranial medial insertion to the medial caudal insertion, passing through the region of the medial suspensory ligament, and this showed to be highly positively correlated with UP, meaning that bulky udders present shorter cleft lengths, with less separation of the halves.

### 3.2. Relationships Between Udder Traits and Intramammary Infections

Udder halves were palpably normal in 57/60 (95%) of the cases, with the absence of clinical signs of mastitis. Three halves from different animals were clinically infected (5%) with milk clots or abnormalities, which made SCC analysis not possible. However, on the samples taken for bacteriology, one was caused by *Staphylococcus aureus* and two by Coagulase-negative staphylococci (CNS). From the 60 halves sampled, 43 were subclinically infected (71.6%). According to the bacteriological milk examination, the animal halves were classified into five groups according to the involved pathogen: Negative (control) (*n* = 9), Yeasts (*n* = 11), *S. aureus* (*n* = 12), Streptococci (*Streptococcus* spp.) (*n* = 12) and CNS *(n* = 11). The overall significance of each studied parameter according to the five different groups is reported in Table 3.

Ushape, Symm, Dsep and Tshape showed significant differences depending on the present microorganism. Teat measures (Tshape and Tangle) had no dependence on the isolated pathogen with regard to the control group. The udder and teat average perimeters of healthy animals (control group) did not reveal significant differences from infected animals. However, the CNS infections were revealed in those parameters to have differences from the other pathogen groups (*p* < 0.05). In fact, only the CNS group differs from the Negative group in terms of UP, UC and DBT (*p* < 0.05). The DBT does not seem to differ according to infected and uninfected animals, except for the CNS group, which showed significant differences for the control group (*p* < 0.001), with lower distance values. Symm did not show significant differences between healthy animals and infected animals. With regard to the UC, we can see that deeper clefts, and therefore a greater separation of the halves, is seen in the CNS group, differently from the control group (*p* < 0.05) and the other pathogens (*p* < 0.001), which show no statistical difference between them.

For the morphological traits subjectively evaluated, Ushape revealed significant differences between the different groups of microorganisms; however, only *S. aureus* was significantly different from the control group (healthy animals). In the evaluation of the Dsep, there were significant differences in the conformation between healthy animals and animals infected with *S. aureus* and *Streptococcus* spp. Tshape was significantly different in animals infected with *Streptococcus* spp. and CNS, compared to healthy animals.

### 3.3. Relationships Between Udder Traits and Somatic Cells

The following groups were established according to the SCC means of the isolated microorganisms: (1) <1300 × 10^3^ SC/mL, (2) >1300 and <6000 × 10^3^ SC/mL, and (3) >6000 × 10^3^ SC/mL. From the studied parameters, the measured UP and UD, and the subjective Ushape, Symm and Dsep, showed significant differences between the SCC established groups (Table 4).

Regarding the subjectively assessed parameters, Symm showed significant differences (*p* < 0.05) between the lowest SCC group and the other groups, with higher counts. The Ushape, the Dsusp and the Dsep revealed significant differences between the group with the lowest SCC and the remaining groups, with higher counts, associated with intramammary infection.

In the parameter-by-parameter analysis (Table 5) and the evaluation of the SCC differences between the different profiles, no differences were observed in the Ushape parameter between the globular and pear-shaped profiles, but differences were observed between these two and the pendular cylindrical shape (*p* < 0.05), with an average of SCC of 9058 × 10^3^ SC/mL. The Dsep showed significant differences in SCC between the slight and severe degrees of separation of the udder halves (*p* < 0.05).

Values are expressed as the mean and standard deviation (SD). Different letters in the same column indicate significant differences between the parameter profiles means, determined by Kruskal–Wallis *H* test (*p* ≤ 0.05).

## 4. Discussion

### 4.1. Udder Conformation

We observed that the studied morphological udder traits are closely related, in agreement with the goats described by [21], and that the increase or decrease in one parameter, such as UP, has an influence on the others, including the teat parameters. As the udder enlarges (increases in perimeter and therefore cleft value), the DBT increases and the teats also increase in size. On the other hand, it gets closer to the tibiotarsal joint (UD) when the teats are closer to the ground (r = 0.6). We may also observe the high variation seen in the minimum and maximum values for udder and teat morphological parameters, indicating a high heterogeneity within the herds.

Both subjective and measured results meet the standard characteristics of the Serrana goat breed, in agreement with [22]. The globular-shaped udder was the most predominant (40%), with slightly separated (56.7%) and symmetrical halves (50%), presenting some degree of suspension (43.3%). The funnel-shaped teat was the most prevalent shape (50%), with an opening of 120° to 160° degrees [20].

The UP does not seem to be related to the lactation number, but to the lactation stage, as well as the DBT and TDG. In agreement with other authors [23], the teat parameters did not have an influence on lactation stage. On the contrary, UD was influenced by lactation number, as animals with more lactations presented longer udders and a greater proximity to the floor. This parameter showed a higher correlation with lactation number rather than with conformational parameters or IMI diagnostic methods.

### 4.2. Relationships Between Udder Traits and Intramammary Infections

The measured conformation parameters UP, UC, TP and the DBT seem to be related to the IMI causative agent. From the subjective traits, Ushape, Dsep and Tshape also demonstrate significant differences depending on the microorganism. Both the UP and TP showed no differences between infected animals and healthy animals. These findings suggest that the measured perimeter by itself is not accurate for diagnostic purposes. Considering our results, the UP seems to be more greatly influenced by the lactation phase, probably due to the increase in volume, while the perimeter of the teats is influenced by the number of lactations, with greater stimulation of milking and kids.

Nevertheless, the highest correlation was obtained between UP and UC (r = 0.89). UC is the vertical measurement of the udder, from the medial cranial insertion to the medial caudal insertion, and it was lower in CNS-infected animals compared to healthy animals, referring to animals with shorter or more attached udders, or with a very severe separation between the halves. The UC’s positive correlation with lactation number and lactation stage indicates that udder dimensions increase as the number of lactations increase, and that udder enlargement results from the physiological response to pregnancy and preparation for lactogenesis and galactopoiesis [21]. The DBT, which is influenced by the increase in the perimeter and stage of lactation, also shows differences between healthy animals (13.9 ± 3.9 cm) and those infected by CNS (9.4 ± 1.8 cm), suggesting that animals with pendulous udders with tightly close vertical teats have higher incidences of IMI. Animals with greater Dsep appear to be more prone to infections caused by *S. aureus* (2.1 ± 0.8)*,* while looser teats seem to have a higher susceptibility to environmental infections.

Ushape manifested differences between healthy animals (1.5 ± 0.8) and animals infected by *S. aureus* (2.5 ± 0.7). Globular and pear-shaped udders are more common in healthy animals, while a pendular cylindrical profile has a higher SCC mean corresponding to infections caused by *S. aureus*. The statistical analysis of SCC in relation to microorganisms reveals significant differences between the control group and *S. aureus*. Despite the higher SCC mean seen in the CNS group, the high variability observed may justify the non-significance in CNS.

### 4.3. Relationships Between Udder Traits and Somatic Cells

The control/negative SCC mean value has been the lowest, in agreement with the values presented by [24].

The measurable parameter, UP, despite presenting significant differences between the different groups of SCC, did not show differences between the lowest cell count group, associated with healthy animals, and the group with the highest SCC, associated with bacterial infection. The significant differences found between SCC groups may be due to the influence of lactation stage and udder fullness [25], revealing that UP may not be a good indicator of udder inflammation.

In the evaluation of the Ushape, there were significant differences in the SCC between the different profiles. We verified the existence of significant differences between the globular (2001.5 ± 3602.8) and pear-shaped (1585.3 ± 2198.1) profiles compared to the pendulous cylindrical profile, this latter being associated with a higher SCC (9058.1 ± 10,250.6). This SCC mean is associated with intramammary infections by bacterial agents, according to the means recorded for each group of isolated microorganisms (Table 3). 

The same happened with the Dsep: the significant differences between the groups of lower and those of higher SCC (Table 4) suggests that the profile corresponding to low counts is the udder with the less pronounced separated halves, and the profile corresponding to a higher SCC mean is the udder with a severe separation of halves (10,452.3 ± 12,934.2), this profile’s mean being associated with bacterial infections (Table 5).

The Dsusp presented significant differences between animals with low SCC and animals with counts above 1300 × 10^3^ SCC/mL. However, in the analysis performed parameter-to-parameter (Table 5), the SCC average of the profiles with lower counts (attached udders) presented higher variations, and averages above 1300 × 10^3^ SCC/mL were compatible with infected animals. The corresponding measurable parameter UD revealed differences between the first group (<1300 × 10^3^ SCC/mL) and the last group (6000 × 10^3^ SCC/mL), meaning that more attached udders present lower SCC [26]. Therefore, the greater the difference is between the ground and the udder floor (UD), the more attached the udder is, and the teats are further from the ground (TDG), thus probably less likely to get injured and infected. However, in the bacteriological analysis, the second group (>1300 and <6000 × 10^3^ SCC/mL) already presented intramammary bacterial infection. This has an influence on the SCC, while not being useful for a presumptive diagnosis, contrary to what was proposed by [26].

In the UP parameter, we can observe a difference between the <1300 × 10^3^ SC/mL group and the >1300 and <6000 × 10^3^ SC/mL group. However, the group with the lowest count, associated with healthy animals (lowest SCC), did not show significant differences from the group with the highest SCC, revealing that the parameter is not a good indicator of udder inflammation.

The UD, and therefore its proximity to the floor, revealed significant differences between the control group and the group with the highest SCC. However, as we know from the bacteriological study, the second group presents most of the time an intramammary bacterial infection.

All other subjective and measurable parameters have not been shown to be related to SCC in milk, and are therefore useless when assessing possible inflammation or intramammary infection.

The Tshape showed significant differences between healthy animals and animals infected by *Streptococcus* spp. and CNS. Animals with conical/funnel teats seem to be more resistant to infections than bottle or balloon shapes. In the analysis of SCC of the Tshape, we did not find significant differences between healthy and infected animals, however these results may be due to the high variability observed in the mean of the profiles. Ref. [27] found that round teat ends had a higher incidence of mastitis, contrary to what was observed in the sharp teats.

Symm showed significant differences between the group of lower SCC and the groups with higher counts, associated with udders with some degree of asymmetry (1.2 ± 0.7). In the parameter-to-parameter comparison (Table 5), however, we found that the means associated with each profile are higher than the established groups for SCC (associated with healthy and infected animals). In this analysis, only the control group (3729.7 ± 9059.3) was different from the group with marked asymmetry (6759.7 ± 5841.4). Adding the fact that there is no significant difference between uninfected animals and animals infected with pathogenic agents, we may think that Symm has an influence on the number of somatic cells, however it does not seem to be related to infectious causes, but instead to apocrine desquamation/secretion, and therefore an increase in epithelial and non-inflammatory cells.

## 5. Conclusions

According to our findings, the most indicative morphological parameters related to IMI incidence are symmetrical globular udders with a slight separation of the halves, and conic teats. The results in general, considering both measurable and subjective parameters, indicate that bifurcated pendular udders, in which the teats are vertically loose and very close to each other, are more likely to have IMI, due to their greater predisposition to suffer injury and contamination.

When evaluating somatic cells in goats, we must keep in mind the high variability existing in the species due to the numerous factors, not only infectious, that condition the results. However, it appears that the udder shape and the degree of separation have a strict connection to SCC.

Therefore, when evaluating the morphological parameters for presumptive diagnose purposes, we should consider not only the breed standard, but also the mentioned factors.

## Figures and Tables

**Table 1:** Descriptive statistics of the udder’s evaluated morphological parameter (*n* = 60).

	Mean ± SD	Range
UP	31.3 ± 9.1	16–54
UC	21.6 ± 8.0	6–38
UD	6.4 ± 4.0	3–15
TP	19.9 ± 7.2	5–38
TL	9.5 ± 3.1	5–20
DBT	11.7 ± 3.4	5–19
TDG	24.3 ± 5.8	11–36
Ushape	1.9 ± 0.8	1–3
Symm	0.7 ± 0.8	0–2
Dsusp	2 ± 0.8	1–3
Dsep	1.6 ± 0.8	1–3
Tshape	1.8 ± 0.9	1–3
Tangle	1.9 ± 0.6	1–3

UP = Udder perimeter (cm); UC = Udder cleft (cm); UD = Udder depth (cm); TP = Teat perimeter (cm); TL = Teat length (cm); DBT = Distance between teats (cm); TDG = Teat distance to the ground (cm); Ushape = Udder shape; Symm = Symmetry; Dsusp = Degree of udder suspension; Dsep = Degree of halves separation; Tshape = Teat shape, Tangle = Teat angle.

**Table 2:** Correlations between mammary measured conformation trait (cm), lactation number and lactation stage in months.

	UP	UC	UD	TP	TL	DBT	TDG
UP	1.000	0.892 **	−0.419 **	0.777 **	0.724 **	0.757 **	−0.640 **
UC	0.892 **	1.000	−0.390 **	0.746 **	0.714 **	0.674 **	−0.613 **
UD	−0.419 **	−0.390 **	1.000	−0.403 **	−0.445 **	−0.416 **	0.611 **
TP	0.777 **	0.746 **	−0.403 **	1.000	0.773 **	0.638 **	−0.660 **
TL	0.724 **	0.714 **	−0.445 **	0.773 **	1.000	0.640 **	−0.743 **
DBT	0.757 **	0.674 **	−0.416 **	0.638 **	0.640 **	1.000	−0.685 **
TDG	−0.640 **	−0.613 **	0.611 **	−0.660 **	−0.743 **	−0.685 **	1.000
Lact n°	NS	0.290 *	−0.596 **	0.426 **	0.333 **	NS	NS
Lact stage	0.392 **	0.381 **	NS	NS	NS	0.562 **	−0.268 *

UP = Udder perimeter; UC = Udder cleft; UD = Udder depth; TP = Teat perimeter; TL = Teat length; DBT = Distance between teats; TDG = Teat distance to the ground; Lact n° = lactation number; Lact stage = lactation stage in months. NS = No significant correlation. * Correlation level of significance 0.05. ** Correlation level of significance 0.01.

**Table 3:** Comparison of udder measured conformation traits (cm) and somatic cell count (SCC) and bacterial status.

Traits	Groups				
	Negative (*n* = 9)	Yeasts (*n* = 11)	*S. aureus* (*n* = 12)	*Streptococcus* spp. (*n* = 12)	CNS (*n* = 11)
UP	30.7 ± 3.7 ^ab^	35.3 ± 6.7 ^a^	34.9 ± 10.5 ^a^	34.4 ± 9.5 ^a^	26.1 ± 5.6 ^b^
UC	20.4 ± 3.8 ^a^	24.9 ± 5.1 ^a^	25.4 ± 6.8 ^a^	26.1 ± 9.9 ^a^	14.4 ± 3.1^b^
TP	19 ± 3.1 ^ab^	20.8 ± 5.8 ^a^	24.2 ± 7.5 ^a^	22.6 ± 8.4 ^a^	17.3 ± 6.4 ^b^
DBT	13.9 ± 3.9 ^a^	13 ± 2.6 ^a^	12.2 ± 2.6 ^a^	11.2 ± 3.2 ^ab^	9.4 ± 1.8 ^b^
Ushape	1.5 ± 0.8 ^a^	1.5 ± 0.5 ^a^	2.5 ± 0.7 ^b^	2 ± 0.9 ^ab^	1.9 ± 1.1 ^a^
Symm	0.6 ± 0.5 ^a^	0.09 ± 0.3 ^b^	0.9 ± 0.8 ^a^	1.1 ± 1 ^a^	0.6 ± 0.5 ^ab^
Dsep	1.3 ± 0.5 ^ac^	1 ± 0 ^a^	2.1 ± 0.8 ^b^	1.9 ± 0.7 ^c^	1.7 ± 1 ^ab^
Tshape	1.1 ± 0.4 ^a^	1.7 ± 1.0 ^a^	1.8 ± 0.6 ^a^	2.2 ± 0.9 ^b^	2.4 ± 1 ^c^
SCC	1218.4 ± 837.2 ^ab^	610.2 ± 259.1 ^a^	4477.5 ± 3605.2 ^c^	4984.9 ± 6680.9 ^cb^	11,322.3 ± 14,866.3 ^cb^

UP = Udder perimeter; TP = Teat perimeter; DBT = Distance between teats; UC = Udder cleft; Ushape = Udder shape; Symm = Symmetry; Dsep = Degree of halves separation; Tshape = Teat shape; SCC = Somatic cell count. Values are expressed as the mean and standard deviation (SD). Different letters (^a,b,c^) in the same line indicate significant differences between the means determined by Kruskal–Wallis *H* test (*p* ≤ 0.05).

**Table 4:** Comparison of udder measured conformation traits (cm) and subjective parameters for SCC groups.

(×10^3^SCC/mL)	<1300	>1300 and <6000	>6000
UP	33.48 ± 6.56 ^a^	28.43 ± 6.2 ^b^	37.6 ± 11.3 ^a^
UD	7.5 ± 3.6 ^a^	6.6 ± 2.9 ^ab^	3.6 ± 4.5 ^b^
Ushape	1.4 ± 0.5 ^a^	2.2 ± 0.9 ^b^	2.5 ± 0.8 ^b^
Sym	0.2 ± 0.4 ^a^	1.2 ± 0.7 ^b^	1.2 ± 0.9 ^b^
Dsusp	1.7 ± 0.6 ^a^	2.2 ± 0.7 ^b^	2.4 ± 0.7 ^b^
Dsep	1.1 ± 0.3 ^a^	2.1 ± 0.6 ^b^	2 ± 0.9 ^b^

UP = Udder perimeter; UD = Udder depth; Ushape = Udder shape; Symm = Symmetry; Dsep = Degree of halves separation; Dsusp = Degree of suspension. Values are expressed as the mean and standard deviation (SD). Different letters (^a,b^) in the same line indicate significant differences between the means determined by Kruskal–Wallis *H* test (*p* < 0.05).

**Table 5:** Association between mammary morphological traits and somatic cells count (10^3^ SC/mL).

Parameters	Profiles	% (*n* = 60)	Mean ± SD of SCC
Symmetry	Symmetrical	50%	3729.7 ± 9059.3 ^a^
	Moderate	30%	3125.6 ± 2852.4 ^ab^
	Asymmetric	20%	6759.7 ± 5841.4 ^b^
Udder shape	Globular	40%	2001.5 ± 3602.8 ^a^
	Pear-shaped	30%	1585.3 ± 2198.1 ^a^
	Cylindrical Pendulous	30%	9058.1 ± 10,250.6 ^b^
Degree separation	Slight	56.7%	2051.3 ± 3681.8 ^a^
	Moderate	26.7%	4618.2 ± 5518.9 ^ab^
	Severe	16.6%	10,452.3 ± 12,934.2 ^b^
Degree suspension	Attached	30%	1561.8 ± 2154.7 ^a^
	Intermediate	43.3%	3128.3 ± 4834.5 ^a^
	Extremely loose	26.6%	8967.3 ± 11,352.4 ^a^
Teat shape	Funnel	50%	3928.2 ± 5237.4 ^a^
	Bottle	23.3%	2842.7 ± 3450.8 ^a^
	Balloon	26.7%	5478.3 ± 10,840.1 ^a^
Teat angle	160°–180°	26.7%	6615.8 ± 11,249.4 ^a^
	120°–160°	43.3%	1632.2 ± 2296.5 ^a^
	90°–120°	30%	5870.1 ± 6356.4 ^a^

Values are expressed as the mean and standard deviation (SD). Different letters in the same column indicate significant differences between the parameter profiles means, determined by Kruskal–Wallis *H* test (*p* ≤ 0.05).
